# Association Between Cholecystectomy and Gastroesophageal Inflammatory Findings After Sleeve Gastrectomy: Endoscopic and Metabolic Evaluation

**DOI:** 10.1007/s11695-026-08947-0

**Published:** 2026-09-26

**Authors:** Gabriel Guerra Cordeiro, Vinícius Vasconcelos do Amaral, Luiz Alberto Reis Mattos Júnior, Flávio Kreimer, José Guido Correia de Araújo Júnior, Álvaro Antônio Bandeira Ferraz

**Affiliations:** Department of Surgery, Federal University of Pernambuco, Recife, Brazil

**Keywords:** Sleeve gastrectomy, Bariatric surgery, Cholecystectomy, Gastroesophageal reflux disease, Bile reflux, Gastritis, Upper gastrointestinal endoscopy

## Abstract

**Introduction:**

Sleeve gastrectomy (SG) is associated with the development or worsening of gastroesophageal reflux disease (GERD). Cholecystectomy following SG — the standard treatment for cholelithiasis resulting from rapid weight loss — may influence GERD pathophysiology by enhancing enterohepatic bile circulation and increasing alkaline reflux.

**Objective:**

To investigate the longitudinal development of endoscopic and histologic esophageal and gastric mucosal findings, alongside systemic metabolic profiles, across defined pre- and post-cholecystectomy intervals in individuals previously managed with SG.

**Methods:**

A longitudinal, retrospective, observational cohort study was conducted with 133 patients who underwent cholecystectomy after SG between 2009 and 2017. Endoscopic findings were analyzed at three time points: pre-SG, pre-cholecystectomy, and post-cholecystectomy.

**Results:**

Compared with the pre-sleeve gastrectomy assessment, moderate esophagitis increased from 3.0% to 12.2% and severe esophagitis from 0.8% to 2.7% at the post-cholecystectomy evaluation. In contrast, the direct comparison between the pre- and post-cholecystectomy assessments demonstrated a significant increase in gastric inflammatory findings, particularly moderate gastritis. Gastritis prevalence increased from 33.9% to 51.4% (*p* < 0.05), with moderate to severe cases rising from 11.9% to 23.0% (*p* < 0.05). BMI and metabolic parameters (HbA1c, insulin, fasting glucose, cholesterol, triglycerides, uric acid, and C-peptide) improved significantly (*p* < 0.001).

**Conclusion:**

Cholecystectomy following sleeve gastrectomy was associated with an increased prevalence and severity of gastritis and a progressive trend in esophagitis over time, without compromising the long-term metabolic benefits of SG.

## Key Points

Progressive upper gastrointestinal inflammatory changes were observed throughout follow-up after sleeve gastrectomy and cholecystectomy.

Gastritis demonstrated the most consistent and statistically significant progression after cholecystectomy.

Metabolic improvements achieved after sleeve gastrectomy were maintained after cholecystectomy.

The observational design does not allow determination of whether these findings are attributable to cholecystectomy or the natural evolution of post-sleeve gastrectomy reflux disease.

## Introduction

 The prevalence of obesity has tripled since 1975 [1]. Given the significant public health impact of this condition and its associated comorbidities, bariatric surgery has become established as an effective treatment for severe obesity, promoting substantial weight loss and meaningful improvement in comorbidities such as diabetes mellitus and systemic arterial hypertension.

Sleeve gastrectomy is the most commonly performed bariatric procedure [2], with satisfactory outcomes and low rates of immediate and late postoperative complications [3]. Anatomical changes inherent to the procedure — flattening of the angle of His, increased intragastric pressure, and greater predisposition to hiatal herniation — favor the onset or worsening of GERD [4, 5].

Rapid and substantial weight loss following SG is associated with a higher incidence of cholelithiasis, occurring in approximately 35% of patients within the first year [6], for which cholecystectomy is the indicated treatment. Gallbladder removal intensifies enterohepatic bile salt circulation, promoting continuous bile flow and alterations in bile composition. These changes may contribute to energy regulation and enhanced weight loss, but also predispose patients to duodenogastric reflux and gastric mucosal injury [7].

Accordingly, this study aimed to evaluate the long-term evolution of esophageal and gastric mucosal findings, assessed through standardized endoscopic and histopathological examinations, in patients who underwent cholecystectomy after sleeve gastrectomy. In addition, longitudinal changes in metabolic parameters were analyzed to explore the interplay between gastrointestinal and metabolic outcomes throughout follow-up.

## Methods

This was a longitudinal, retrospective, observational cohort study. A total of 234 adult patients who underwent cholecystectomy following SG at the Hospital das Clínicas of the Federal University of Pernambuco (HC/UFPE-EBSERH) and at Hospital Esperança Recife, Brazil, between 2009 and 2017 were initially screened. The final sample comprised 133 patients.

The study population comprised adult patients (aged > = 18 years) with a baseline body mass index (BMI) > = 35 kg/m² who underwent standardized primary laparoscopic sleeve gastrectomy between 2009 and 2017, and subsequently required a secondary laparoscopic cholecystectomy due to symptomatic, ultrasound-proven cholelithiasis. To be eligible for final study inclusion, patients were strictly required to have complete, paired clinical, metabolic, and upper gastrointestinal endoscopic data—including mandatory histological reports from gastric and esophageal biopsies—across three explicit chronological milestones: [1] Pre-SG baseline evaluation [2], Pre-cholecystectomy intermediate baseline evaluation, and [3] Post-cholecystectomy final long-term follow-up evaluation. Patients with a prior history of gastric resections, antireflux surgery, underlying foregut malignancies, or incomplete longitudinal datasets were excluded from analysis. The final sample comprised 133 patients.

Surgical procedures were executed using standard techniques. Primary SG was performed over a calibration bougie (ranging from 36 F to 38 F) with the gastric transection initiated approximately 4 to 5 cm proximal to the pylorus and carried upwards linearly to the angle of His. Crucial attention was directed toward clearing the short gastric vessels and ensuring the complete resection of the gastric fundus. Concomitant hiatal hernias, when identified macroscopically during the primary operation, were repaired via posterior cruroraphy. Secondary cholecystectomies were conducted laparoscopically via a standard four-port technique, achieving a clear view of safety before cystic duct and artery ligation.

High-resolution upper gastrointestinal endoscopies were performed routinely at the specified intervals by experienced endoscopists. Esophagitis severity was rigorously categorized according to the standardized Los Angeles (LA) Classification (Grades A, B, C, and D). During each endoscopic evaluation, systematic mucosal tissue biopies were harvested from the esophageal mucosa, the gastric body, and the antrum. Tissue specimens were fixed in 10% buffered formalin, stained with hematoxylin-eosin (H&E), and evaluated by dedicated gastrointestinal pathologists to confirm the presence and severity of chronic gastritis, follicular gastritis, atrophic mucosal changes, intestinal metaplasia, glandular dysplasia, or Barrett’s esophagus. The presence of Helicobacter pylori was systematically sought via Giemsa staining.

Venous blood samples were collected after a mandatory 12-hour fast at each of the three defined study milestones. Laboratory assays quantified fasting plasma glucose, hemoglobin A1c (HbA1c), fasting serum insulin, C-peptide, total cholesterol, high-density lipoprotein cholesterol (HDL-c), low-density lipoprotein cholesterol (LDL-c), serum triglycerides, uric acid, and serum iron. Homeostatic Model Assessment of Insulin Resistance (HOMA-IR) was mathematically derived via standard formulas.

Statistical analysis was performed using IBM SPSS v18. Categorical variables were analyzed with Pearson’s chi-square or Fisher’s exact test. The Friedman test was used for repeated-measures comparisons. Data normality was assessed using the Shapiro-Wilk test. Because endoscopic findings represented ordinal variables and several continuous variables demonstrated non-normal distributions, repeated-measures analyses were performed using the Friedman test, which is the appropriate non-parametric alternative to repeated-measures ANOVA for related samples assessed across more than two time points. Statistical significance was set at *p* < 0.05.

## Results

### Demographic Profile and Postoperative Follow-up

The sample comprised 133 patients, predominantly female (82.7%), aged 30–45 years (47.4%). Prior abdominal surgery was reported in 36.1%. Most patients reported no alcohol use or smoking (62.9%). The most prevalent interval between SG and cholecystectomy was 12–24 months (44.4%), and post-cholecystectomy follow-up exceeded 12 months in 62.1% of cases. (Table 1)

**Table 1:** Distribution of demographic profile, comorbidities, and lifestyle habits of patients who underwent sleeve gastrectomy + cholecystectomy

Assessed Factors	*n*	%	*p*-value
Age			
16 - 29 years	50	37,6	<0,001¹
30 - 45 years	63	47,4	
46 - 59 years	17	12,8	
60 years or older	3	2,3	
Sex			
Male	23	17,3	<0,001¹
Female	110	82,7	
Comorbidities			
SAH	11	18,0	<0,001¹
DM	3	4,9	
Dyslipidemia	5	8,2	
Prior abdominal surgery	22	36,1	
Family history of cancer	15	24,6	
Other	5	8,2	
Lifestyle Habits			
Alcohol use	39	29,5	<0,001¹
Smoking	1	0,8	
Alcohol use and smoking	9	6,8	
No alcohol use and no smoking	83	62,9	

SAH: Systemic arterial hypertension; DM: Diabetes mellitus. ¹p-value from Pearson’s chi-square test

### Postoperative follow-up

Table 2 shows the interval between sleeve gastrectomy (SG) and cholecystectomy, as well as the postoperative follow-up period after cholecystectomy. A statistically significant higher prevalence was observed for an interval of 12 to 24 months between SG and cholecystectomy (44.4%), as well as for a post-cholecystectomy follow-up period exceeding 12 months (62.1%). (Table 2)

**Table 2:** Distribution of the interval between procedures (Sleeve Gastrectomy and Cholecystectomy) and post-cholecystectomy follow-up period

Assessed Factor ±	*N*. patients	%	Mean (months)	SD	Min.	Max	*P* value
Interval between SG and Cholecystectomy							<0,001
0 – 6 months	9	6,7%	5,1	±0,9	4	6	
> 6-12 months	36	27,1%	9,3	±2,0	7	12	
> 12-24 months	59	44,4%	16,8	±2,9	13	24	
> 24 months	29	21,8%	46,4	±30,1	25	161	
Post-Cholecystectomy Follow-up Period							<0,001
0–6 months	41	31,1%	1,6	±1,7	0,3	6	
> 6-12 months	9	6,8%	9,6	±1,1	8	11	
> 24 months	82	62,1%	40,8	±17,5	15	92	

¹ *p*-value from Pearson’s chi-square test

### BMI Analysis

Median BMI was 39.96 kg/m² at pre-SG, decreasing to 27.84 kg/m² at pre-cholecystectomy and remaining at 27.84 kg/m² at post-cholecystectomy. A statistically significant difference in BMI distribution was identified across assessment time points (Table 3).

**Table 3:** BMI analysis of evaluated patients according to time point of assessment

Assessment Time Point				
	Pré-operatório		Pós-operatório	
Assessed Measures	Pre-SG	Pre-Cholecystectomy	Post-Cholecystectomy	*p*-value
Minimum	34,93	20,42	20,83	
Maximum	53,11	42,43	46,70	<0,001¹
Median	39,96	27,84	27,84	
Interquartile range	4,20	4,13	5,24	

¹*p*-value from Friedman test

### Esophageal and Gastric Endoscopic Findings

Between the pre-SG and pre-cholecystectomy time points (Table 4), mild esophagitis decreased from 38.3% to 22.9%, while moderate esophagitis increased from 3.0% to 8.3% and severe esophagitis from 0.8% to 1.8%. Barrett’s esophagus appeared at the pre-cholecystectomy time point (0.9%). No statistical significance was found in the esophageal analysis at this comparison. In the gastric segment, a statistically significant reduction in mild gastritis (49.6% to 22.0%) and increase in moderate gastritis (5.3% to 11.9%) were identified.

Comparing pre-SG and post-cholecystectomy time points (Table 5), statistically significant increases in moderate esophagitis (3.0% to 12.2%) and severe esophagitis (0.8% to 2.7%) were found, with one case of Barrett’s esophagus (1.3%). In the gastric segment, mild gastritis decreased (49.6% to 28.4%) and moderate gastritis increased considerably (5.3% to 23.0%), both with statistical significance.

Between the pre- and post-cholecystectomy time points (Table 6), no statistical significance was found in the esophageal segment. In the gastric segment, incidences of mild (22.0% to 28.4%) and moderate gastritis (11.9% to 23.0%) increased significantly.

**Table 4:** Comparative analysis of endoscopic classification of the esophageal and gastric segments between the pre-SG and pre-cholecystectomy time points

Assessed Findings	Evaluation moment			*p*-value
	Pre-SG	Pre-Cholecystectomy	Pos-Cholecystectomy	
Esophagus				
Normal	77(57,9%)	72(66,1%)	44(59,5%)	0,194¹0,081²
Mild	51(38,3%)	25(22,9%)	18(24,3%)	
Moderate	4(3,0%)	9(8,3%)	9(12,2%)	
Severe	1(0,8%)	2(1,8%)	2(2,7%)	
Barrett’s esophagus	-	1(0,9%)	1(1,3%)	
Stomach				
Normal	59(44,4%)	72(66,1%)	36(48,6%)	<0,001¹0,551²
Mild	66(49,6%)	24(22,0%)	21(28,4%)	
Moderate	7(5,3%)	13(11,9%)	17(23,0%)	
Severe	1(0,7%)	-	-	

¹*p*-value from Pearson’s chi-square test (Normal vs. Mild, Moderate, Severe, and Barrett’s*)

²*p*-value from Pearson’s chi-square test (Normal vs. Moderate, Severe, and Barrett’s*)

*Applies to the esophageal segment only

**Table 5:** Comparative analysis of endoscopic classification of the esophageal and gastric segments between the pre-SG and post-cholecystectomy time points

Exames avaliados	Evaluation moment			p-valor
	Pre-SG	Pre-Cholecystectomy	Pos-Cholecystectomy	
Esophagus				0,874¹ 0,007²
Normal	77(57,9%)	72(66,1%)	44(59,5%)	
Mild	51(38,3%)	25(22,9%)	18(24,3%)	
Moderate	4(3,0%)	9(8,3%)	9(12,2%)	
Severe	1(0,8%)	2(1,8%)	2(2,7%)	
Barrett’s esophagus	-	1(0,9%)	1(1,3%)	
Stomach				0,552¹ 0,006²
Normal	59(44,4%)	72(66,1%)	36(48,6%)	
Mild	66(49,6%)	24(22,0%)	21(28,4%)	
Moderate	7(5,3%)	13(11,9%)	17(23,0%)	
Severe	1(0,7%)	-	-	

¹*p*-value from Pearson’s chi-square test (Normal vs. Mild, Moderate, Severe, and Barrett’s*)

²*p*-value from Pearson’s chi-square test (Normal vs. Moderate, Severe, and Barrett’s*)

*Applies to the esophageal segment only

**Table 6:** Comparative analysis of endoscopic classification of the esophageal and gastric segments between the pre-cholecystectomy and post-cholecystectomy time points

Assessed findings	Evaluation moment			*p*-value
	Pre-SG	Pre-Cholecystectomy	Pos-Cholecystectomy	
Esophagus				
Normal	77(57,9%)	72(66,1%)	44(59,5%)	
Mild	51(38,3%)	25(22,9%)	18(24,3%)	0,363¹
Moderate	4(3,0%)	9(8,3%)	9(12,2%)	0,271²
Severe	1(0,8%)	2(1,8%)	2(2,7%)	
Barrett’s esophagus	-	1(0,9%)	1(1,3%)	
Stomach				
Normal	59(44,4%)	72(66,1%)	36(48,6%)	
Mild	66(49,6%)	24(22,0%)	21(28,4%)	0,018¹
Moderate	7(5,3%)	13(11,9%)	17(23,0%)	0,020²
Severe	1(0,7%)	-	-	

¹*p*-value from Pearson’s chi-square test (Normal vs. Mild, Moderate, Severe, and Barrett’s*)

²*p*-value from Pearson’s chi-square test (Normal vs. Moderate, Severe, and Barrett’s*)

*Applies to the esophageal segment only

### Laboratory Measures

Table 7 presents the mean and standard deviation of laboratory measures. HbA1c, insulin, fasting glucose, total cholesterol, LDL, triglycerides, uric acid, C-peptide, and neutrophil-to-lymphocyte ratio were all higher at pre-SG and improved significantly at subsequent time points (*p* < 0.001). Vitamin B12 and iron showed highest mean values at pre-cholecystectomy. Zinc and albumin showed no statistically significant variation across time points (*p* = 0.658 and *p* = 0.215, respectively) (Table 7).

**Table 7:** Mean and standard deviation of laboratory measures according to the assessment time point

Laboratory Parameter	Evaluation moment			*p*-value
	Pre-SG	Pre-Cholecystectomy	Pos-Cholecystectomy	
Glycated hemoglobin	5,61±0,52	5,24±0,58	5,26±0,64	<0,001¹
Insulin	23,50±13,97	8,02±5,34	8,10±3,66	<0,001¹
Fasting glucose	98,12±71,07	84,08±7,80	85,83±7,01	<0,001¹
Total cholesterol	197,55±34,68	186,74±31,42	194,26±33,70	0,010¹
LDL cholesterol	121,36±29,35	114,38±26,19	114,91±27,90	0,017¹
Triglycerides	137,63±61,64	86,59±31,91	93,85±51,47	<0,001¹
Uric acid	5,33±1,34	4,52±1,12	4,23±1,04	<0,001¹
C-peptide	3,38±1,15	1,88±0,71	1,98±0,95	<0,001¹
Neutrophil-to-lymphocyte ratio	2299,33±581,68	2064,33±660,22	1972,85±527,94	<0,001¹
Zinc	87,98±17,35	85,09±16,48	86,32±14,31	0,658¹
Vitamin B12	451,03±185,30	506,01±209,15	437,80±158,26	0,001¹
Iron	83,57±29,44	100,65±35,13	94,93±29,91	0,021¹
Albumin	4,35±0,26	4,29±0,28	4,38±0,29	0,215¹

¹*p*-value from Friedman test

## Discussion

### Sleeve Gastrectomy and GERD

Gastroesophageal reflux disease occurs in 15–20% of patients who undergo SG, with onset, persistence, and worsening of preoperative symptoms, reflecting the heterogeneity of clinical responses to the procedure [8]. Demographic and metabolic factors have been associated with an increased risk of GERD following SG, including female sex, older age, white race, and tobacco use, suggesting that individual characteristics influence the pathophysiology of reflux after the procedure [4].

The longitudinal endoscopic findings observed in this cohort reinforce the complex relationship between sleeve gastrectomy and reflux-related disease. Although esophageal inflammatory changes progressed over time, the magnitude and consistency of gastric inflammatory findings were more pronounced. These observations support previous evidence indicating that anatomical and physiological alterations following sleeve gastrectomy may influence both esophageal and gastric mucosal integrity during long-term follow-up. In the gastric segment, the influence of SG on the inflammatory pattern of the mucosa is evident. In the comparison between the pre-SG and pre-cholecystectomy time points (Table 4), a statistically significant increase in the prevalence of moderate gastritis was observed, with further progression at the post-cholecystectomy time point (Table 5).

In agreement with the literature, these findings suggest that anatomical modifications — such as reduced gastric volume, increased intraluminal pressure, loss of fundic compliance, and possible alteration of the angle of His — play a central role in the antireflux mechanism [9]. Da Silva et al. reported a significant increase in both the prevalence and severity of GERD after sleeve gastrectomy, reinforcing that, despite its metabolic and weight-related benefits, SG influences gastroesophageal reflux, which must be considered in patient selection and follow-up [10].

A particularly relevant finding was the progressive increase in gastric inflammatory changes throughout follow-up. Notably, this process appeared to begin before cholecystectomy, suggesting that sleeve gastrectomy itself may contribute to chronic gastric mucosal injury. Altered antropyloric physiology, accelerated gastric emptying, changes in gastric motility, and increased exposure of the gastric remnant to duodenal contents have all been proposed as potential mechanisms [11]. Following cholecystectomy, continuous bile flow may further amplify these processes, creating a multifactorial environment that favors persistent low-grade inflammation. Therefore, the progression of gastritis observed in this cohort is likely the result of several interacting physiological mechanisms rather than a consequence of a single surgical intervention.

A single case of Barrett’s esophagus was identified during follow-up. Although no meaningful conclusions can be drawn from an isolated finding, Barrett’s esophagus remains clinically relevant because it represents a recognized long-term complication of chronic gastroesophageal reflux after sleeve gastrectomy [12], reinforcing the importance of long-term endoscopic surveillance in selected patients with persistent reflux-related findings.

### Cholecystectomy and GERD

Cholecystectomy has been hypothesized to favor continuous bile flow and may contribute to duodenogastric reflux [7]. Because bile reflux was not directly measured in the present study, the proposed mechanistic relationship between cholecystectomy, bile exposure, gastritis, and GERD should be interpreted as hypothetical. The increased circulation of bile salts in the intestinal lumen is related to the modulation of metabolic pathways, with influence on energy physiology and gastrointestinal motility [13]. Continuous bile flow predisposes to duodenogastric reflux and greater exposure of the gastric mucosa to bile acids, creating a favorable environment for mixed, both acid and alkaline, reflux [14]. Changes in gastrointestinal hormone levels — including cholecystokinin and motilin — have been associated with an increased frequency of transient lower esophageal sphincter relaxations, a central mechanism in GERD pathophysiology [7].

Alkaline reflux, characterized by the presence of bile and pancreatic secretions in the stomach and esophagus, is associated with persistent mucosal inflammation, direct epithelial injury, and activation of chronic pro-inflammatory pathways. Hydrophobic bile acids, such as deoxycholic acid, cause oxidative DNA damage and activate NF-kB — the primary inflammatory pathway associated with biliary reflux — leading to increased expression of cytokines IL-6, IL-8, TNF-α, and COX-2 [14, 15]. Prolonged exposure of the esophageal mucosa to bile acids, particularly when combined with gastric acid and reactive oxygen species-mediated oxidative stress, potentiates cellular injury and has been linked to the development of more severe esophagitis, intestinal metaplasia, and, in advanced cases, dysplasia [14, 15].

In the present study, progressive upper gastrointestinal inflammatory changes were observed throughout follow-up. However, because no control group of sleeve gastrectomy patients without cholecystectomy was available, it is not possible to determine whether the observed progression reflects the natural history of post-sleeve reflux disease, the effects of cholecystectomy, or the interaction of both processes. Notably, the most robust and statistically significant finding after cholecystectomy was the increase in gastric inflammatory changes, whereas esophageal findings showed only a non-significant trend toward progression. Analysis of Table 6 demonstrated that, despite the absence of statistically significant differences in esophageal endoscopic classifications between the pre- and post-cholecystectomy periods, there was significant worsening of inflammatory findings in the gastric segment, characterized by increased incidence of mild and moderate gastritis following gallbladder removal. This pattern may be consistent with an early predominance of gastric mucosal changes, though whether this reflects a specific effect of cholecystectomy or the continuing natural history of post-sleeve gastrectomy disease cannot be determined from the present data.

These findings corroborate evidence that cholecystectomy modifies bile flow dynamics and gastrointestinal physiology, favoring greater exposure of the gastric mucosa to bile acids — particularly in the antrum and gastric body — thereby promoting low-to-moderate-grade chronic inflammation [16]. In patients who have previously undergone sleeve gastrectomy, this exposure may be potentiated by the reduction in gastric volume and altered motility, leading to more frequent and prolonged contact between bile and the residual mucosa [17].

An additional factor that may contribute to duodenogastric reflux after sleeve gastrectomy is the alteration of gastric motility resulting from partial antral resection. Changes in gastric emptying dynamics and pyloric function may influence the exposure of the gastric remnant to duodenal contents, potentially interacting with post-cholecystectomy bile flow changes. Therefore, the observed gastric inflammatory findings are likely multifactorial rather than exclusively attributable to cholecystectomy [9, 11, 18].

### Cholecystectomy and Metabolic Effects

The increase in bile acid levels in the enterohepatic circulation following cholecystectomy influences the activation of the TGR5 receptor and stimulates the secretion of incretin peptides, particularly glucagon-like peptide-1 (GLP-1). This mechanism is directly associated with improved glycemic control, reduced glycated hemoglobin levels, and improved insulin sensitivity — metabolic benefits also observed in the post-sleeve gastrectomy period [13].

In the present study, despite the impact of cholecystectomy on GERD, post-SG clinical and metabolic outcomes were preserved. Significant weight loss was maintained, with sustained improvement in laboratory parameters, including glycated hemoglobin, insulin sensitivity, reduction in systemic inflammatory state, C-peptide, lipid profile, neutrophil-to-lymphocyte ratio, and uric acid — findings that corroborate the benefits widely attributed to SG [19]. These results reinforce that cholecystectomy does not compromise the metabolic efficacy of SG, although it may influence gastroesophageal functional outcomes [20].

The findings of the present study suggest that patients undergoing cholecystectomy after SG require individualized surgical planning to optimize functional and metabolic outcomes [21]. Future prospective studies, including randomized clinical trials and detailed physiological assessments of GERD pathophysiology, are needed to substantiate these findings and elucidate the underlying mechanisms [15]. From a clinical perspective, these findings underscore the importance of long-term gastrointestinal surveillance in patients undergoing sleeve gastrectomy, particularly among those who subsequently require cholecystectomy [22]. While the metabolic benefits of bariatric surgery appear to be preserved, progressive inflammatory changes within the upper gastrointestinal tract may warrant closer endoscopic monitoring and individualized management strategies.

### Limitations

Several limitations should be acknowledged. First, the retrospective observational design precludes causal inference. Second, no control group of sleeve gastrectomy patients who did not undergo cholecystectomy was available, preventing differentiation between the natural progression of post-sleeve reflux disease and the potential contribution of cholecystectomy. Third, GERD assessment relied exclusively on endoscopic findings, without symptom scores, validated reflux questionnaires, pH monitoring, impedance testing, or Bilitec evaluation. Fourth, important confounders such as hiatal hernia progression, proton pump inhibitor use, smoking status during follow-up, sleeve anatomy, NSAID use, Helicobacter pylori infection, and gastric bile staining were not systematically available. Finally, the anatomical distribution of gastritis and objective markers of bile reflux were not recorded.

## Conclusion

In this longitudinal retrospective cohort of patients who underwent cholecystectomy after sleeve gastrectomy, progressive upper gastrointestinal inflammatory changes were observed, characterized most consistently by a significant increase in the prevalence and severity of gastritis, whereas esophageal findings demonstrated a non-significant trend toward worsening. These observational findings must be interpreted in light of key methodological limitations, notably the retrospective design, the absence of a control group of sleeve gastrectomy patients without cholecystectomy, and the lack of objective functional reflux measurements (such as pH-impedance or bile reflux evaluation).

Consequently, the data cannot determine whether the observed endoscopic progression is directly attributable to cholecystectomy or reflects the natural history of post-sleeve reflux disease. While long-term metabolic benefits—including weight loss and improvements in glycemic and lipid profiles—were safely preserved following gallbladder removal, the progressive mucosal inflammation highlights the clinical importance of implementing long-term endoscopic surveillance and individualized follow-up strategies. Future prospective controlled studies incorporating objective functional evaluations are required to clarify these associations.

## Data Availability

No datasets were generated or analysed during the current study.

## References

[1] World Health Organization. Obesity and overweight. Geneva: WHO; 2020.

[2] Vitiello A, Abu-Abeid A, Dayan D, Berardi G, Musella M. Long-Term Results of Laparoscopic Sleeve Gastrectomy: a Review of Studies Reporting 10 + Years Outcomes. Obes Surg. 2023;33(11):3565–70. 10.1007/s11695-023-06824-8 PMC10603075 37743393

[3] International Federation for the Surgery of Obesity and Metabolic Disorders (IFSO). Ninth IFSO global registry report. Liem R, editor. IFSO; 2025 [cited 2026 Jun 3]. Available from: https://www.ifso.com/ifso-registry.php

[4] Bevilacqua LA, et al. Incidence of GERD, esophagitis, Barrett’s esophagus, and esophageal adenocarcinoma after bariatric surgery. Surg Obes Relat Dis. 2020;16(11):1828–36. 10.1016/j.soard.2020.06.016 32711954

[5] Yeung KTD, Penney N, Ashrafian L, Darzi A, Ashrafian H. Does Sleeve Gastrectomy Expose the Distal Esophagus to Severe Reflux? A Systematic Review and Meta-analysis. Ann Surg. 2020;271(2):257–65. 10.1097/SLA.0000000000003275 30921053

[6] Sneineh MA, et al. Increased Incidence of Symptomatic Cholelithiasis After Bariatric Roux-En-Y Gastric Bypass. Obes Surg. 2020;30(3):846–50. 10.1007/s11695-019-04366-6 31901127

[7] Lin OS, et al. The association between cholecystectomy and gastroesophageal reflux symptoms: a prospective controlled study. Ann Surg. 2010;251(1):40–5. 10.1097/SLA.0b013e3181b9eca4 PMC3676300 19858706

[8] Manifold DK, et al. Effect of cholecystectomy on gastroesophageal and duodenogastric reflux. Am J Gastroenterol. 2000;95(10):2746–50. 10.1111/j.1572-0241.2000.02298.x 11051343

[9] Johari Y, Lim G, Wickremasinghe A, Yue H, Seah J, Ooi G, Playfair J, Laurie C, Beech P, Yap K, Hebbard G, Brown W, Burton P. Pathophysiological Mechanisms of Gastro-esophageal Reflux After Sleeve Gastrectomy. Ann Surg. 2022;276(5):e407–16. 10.1097/SLA.0000000000004637 33214478

[10] da Silva JTD, Santa-Cruz F, Cavalcanti JMS, et al. Incidence of Abnormalities of the Gastric Tube Following Sleeve Gastrectomy and Its Role on Esophagitis Progression. Obes Surg. 2023;33:263–7. 10.1007/s11695-022-06375-4 36460942

[11] Ardila-Hani A, Soffer EE. Review article: the impact of bariatric surgery on gastrointestinal motility. Aliment Pharmacol Ther. 2011;34(8):825–31. 10.1111/j.1365-2036.2011.04812.x 21854401

[12] Qumseya BJ, Qumsiyeh Y, Ponniah SA, Estores D, Yang D, Johnson-Mann CN, Friedman J, Ayzengart A, Draganov PV. Barrett’s esophagus after sleeve gastrectomy: a systematic review and meta-analysis. Gastrointest Endosc. 2021;93(2):343–e3522. 10.1016/j.gie.2020.08.008 32798535

[13] de Lucena AVS, et al. Cholecystectomy Concomitant with Bariatric Surgery: Safety and Metabolic Effects. Obes Surg. 2022;32(4):1093–102. 10.1007/s11695-022-05889-1 35064462

[14] Vaezi MF, Richter JE. Role of acid and duodenogastroesophageal reflux in gastroesophageal reflux disease. Gastroenterology. 1996;111(5):1192–9. 10.1053/gast.1996.v111.pm8898632 8898632

[15] Souza RF, et al. Gastroesophageal reflux might cause esophagitis through a cytokine-mediated mechanism rather than caustic acid injury. Gastroenterology. 2009;137(5):1776–84. 10.1053/j.gastro.2009.07.055 19660463

[16] Serra FE, Cohen RV. Gastroesophageal reflux disease after sleeve gastrectomy. Dig Med Res. 2024;7:5.

[17] Eldredge TA, et al. Once in a Bile - the Incidence of Bile Reflux Post-Bariatric Surgery. Obes Surg. 2022;32(5):1428–38. 10.1007/s11695-022-05977-2 PMC8883020 35226339

[18] Shah Gilani SN, Bass GA, Kharytaniuk N, Downes MR, Caffrey EF, Tobbia I, Walsh TN. Gastroesophageal Mucosal Injury after Cholecystectomy: An Indication for Surveillance? J Am Coll Surg. 2017;224(3):319–26. 10.1016/j.jamcollsurg.2016.12.003 27993699

[19] Peterli R, et al. Effect of Laparoscopic Sleeve Gastrectomy vs Laparoscopic Roux-en-Y Gastric Bypass on Weight Loss. JAMA. 2018;319(3):255–65. 10.1001/jama.2017.20897 PMC5833546 29340679

[20] Ferraz ÁAB, et al. The Impact of Cholecystectomy on the Metabolic Profile of Patients Previously Submitted to Bariatric Surgery. Surg Laparosc Endosc Percutan Tech. 2025;35(1):e1348. 10.1097/SLE.0000000000001348 39618187

[21] Mercan E, et al. Cholecystectomy and duodenogastric reflux: interacting effects over the gastric mucosa. Springerplus. 2016;5(1):1970. 10.1186/s40064-016-3641-z PMC5108731 27917345

[22] Brown WA, Johari Halim Shah Y, Balalis G, Bashir A, Ramos A, Kow L, Herrera M, Shikora S, Campos GM, Himpens J, Higa K. IFSO Position Statement on the Role of Esophago-Gastro-Duodenal Endoscopy Prior to and after Bariatric and Metabolic Surgery Procedures. Obes Surg. 2020;30(8):3135–53. 10.1007/s11695-020-04720-z 32472360

